# Supplementary material for: Quantifying Cross-Modal Association Confidence for Single-Cell RNA-ATAC Integration
Source: bioRxiv. 2026 May 12:2026.05.07.723400. Preprint. [Version 1] doi: 10.64898/2026.05.07.723400 (PMC13192985; doi:10.64898/2026.05.07.723400)
Supplement: 2 [file NIHPP2026.05.07.723400v1-supplement-2.pdf]

bioRxiv preprint doi: https://doi.org/10.64898/2026.05.07.723400; this version posted May 12, 2026. The copyright holder for this preprint (which was not certified by peer review) is the author/funder, who has granted bioRxiv a license to display the preprint in perpetuity. It is made available under a CC-BY 4.0 International license.

Additional Files

Supplementary Figures:

- Supplementary Figure S1: Improvement in each metric from the benchmark using paired data.
- Supplementary Figure S2: Improvement in each metric from the benchmark using unpaired data.
- Supplementary Figure S3: Improvement in each metric from the benchmark using spatial data.

Supplementary Tables:

- Supplementary Table S1: List of benchmarking datasets
- Supplementary Table S2: List of ENCODE human datasets used to compute CLIC scores and their sequencing metrics
- Supplementary Table S3: List of ENCODE mouse datasets used to compute CLIC scores and their sequencing metrics

bioRxiv preprint doi: https://doi.org/10.64898/2026.05.07.723400; this version posted May 12, 2026. The copyright holder for this preprint (which was not certified by peer review) is the author/funder, who has granted bioRxiv a license to display the preprint in perpetuity. It is made available under a CC-BY 4.0 International license.

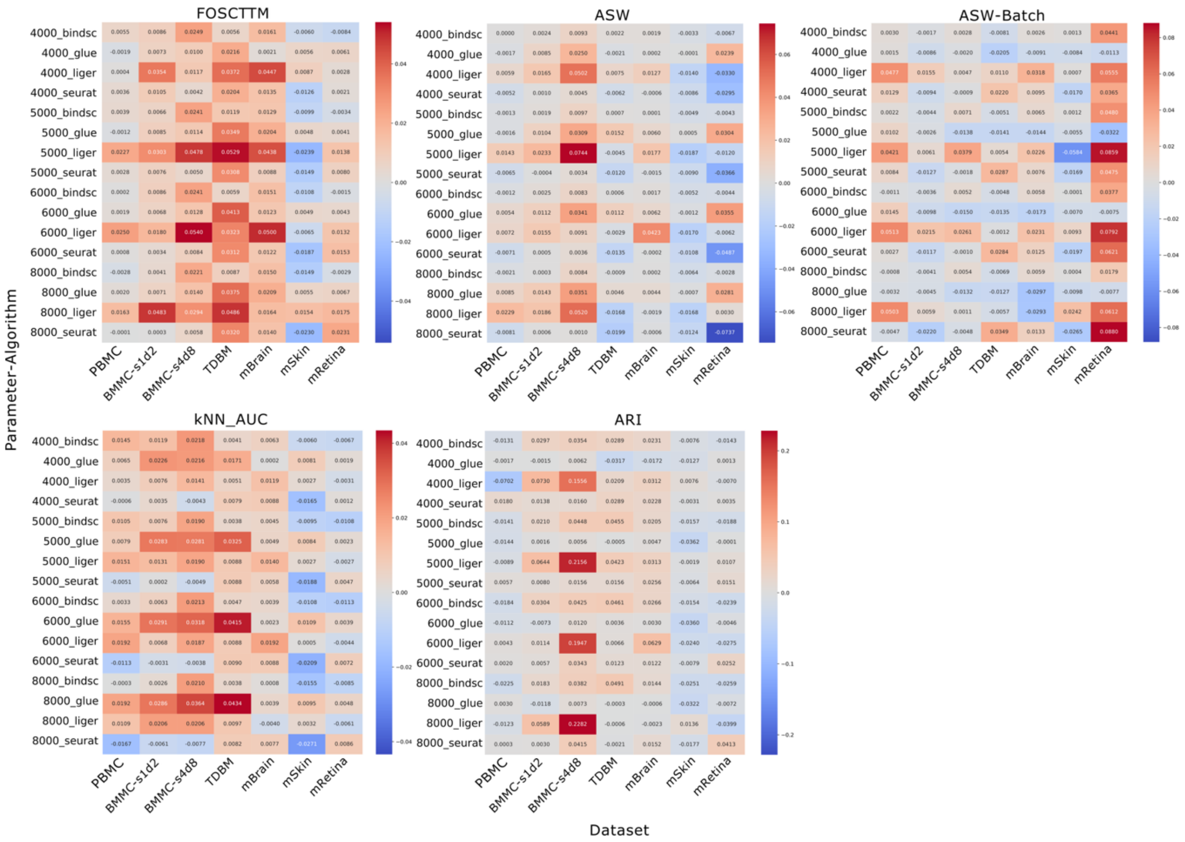

Fig. S1 Improvement in each metric from benchmarking experiment using paired data. Each row of the heatmap represents a combination of the parameter *m* for CLIC_m and the integration algorithm used. Each column represents the dataset. Red represents improvement over baseline.

bioRxiv preprint doi: https://doi.org/10.64898/2026.05.07.723400; this version posted May 12, 2026. The copyright holder for this preprint (which was not certified by peer review) is the author/funder, who has granted bioRxiv a license to display the preprint in perpetuity. It is made available under a CC-BY 4.0 International license.

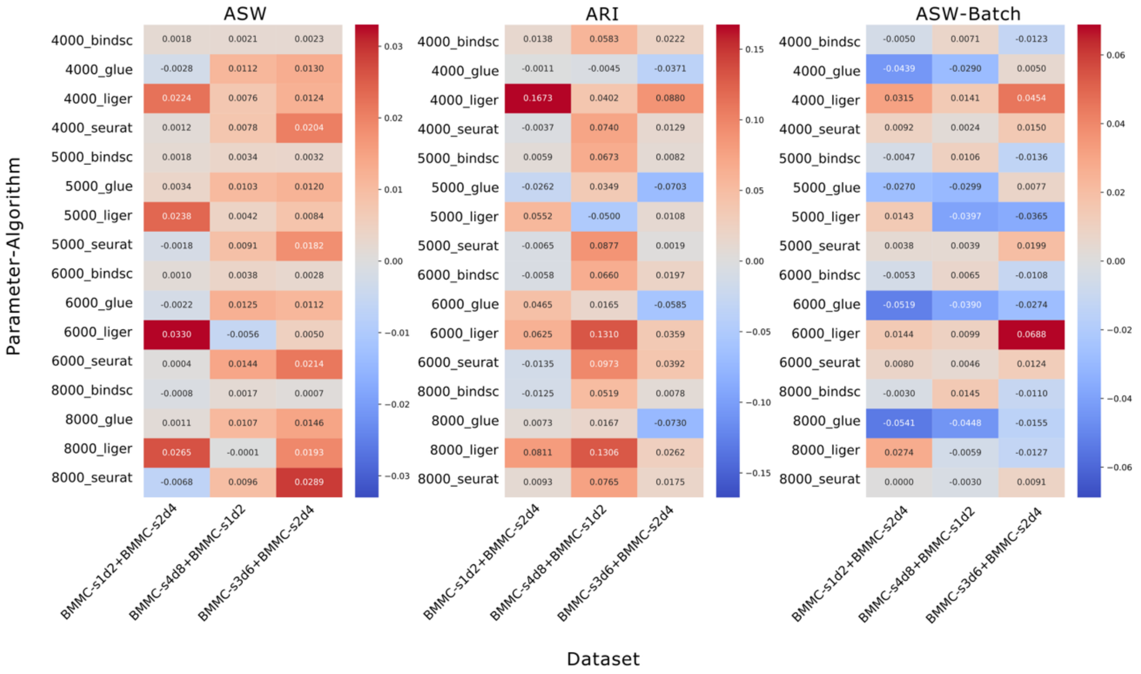

Fig. S2 Improvement in each metric from benchmarking experiment using unpaired BMMC data. Each row of the heatmap represents a combination of the parameter *m* for CLIC_m and the integration algorithm used. Each column represents the pair of batches used (RNA+ATAC). Red represents improvement over baseline.

bioRxiv preprint doi: https://doi.org/10.64898/2026.05.07.723400; this version posted May 12, 2026. The copyright holder for this preprint (which was not certified by peer review) is the author/funder, who has granted bioRxiv a license to display the preprint in perpetuity. It is made available under a CC-BY 4.0 International license.

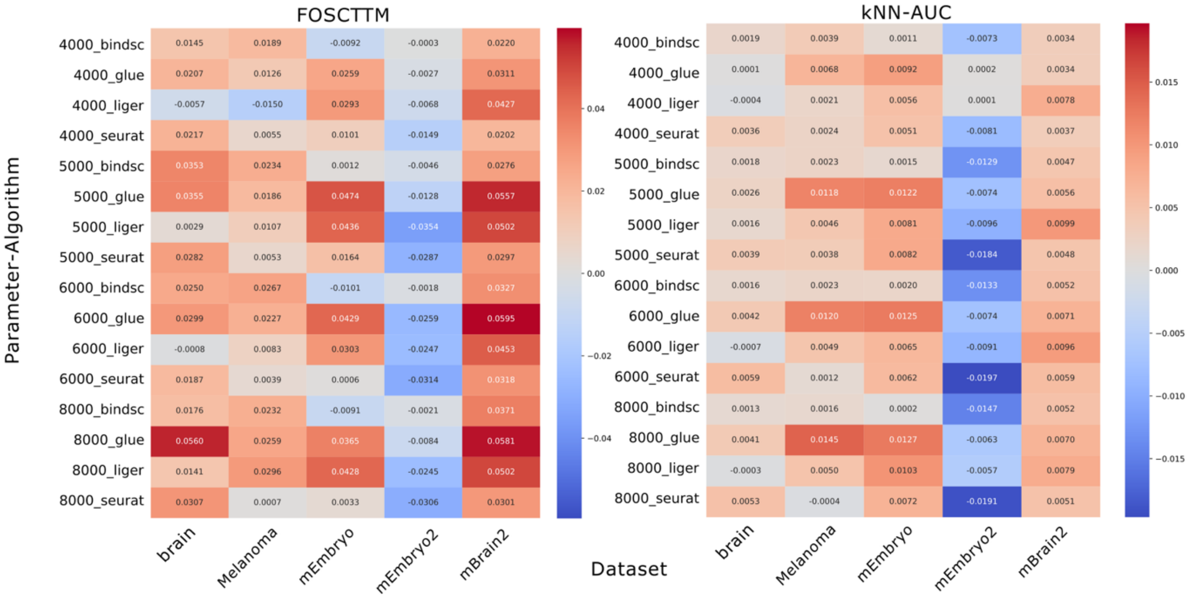

Fig. S3 Improvement in each metric from benchmarking experiment using spatial multiome data. Each row of the heatmap represents a combination of the parameter *m* for CLIC_m and the integration algorithm used. Each column represents the spatial dataset. Red represents improvement over baseline.
